# Stable semiconductor black phosphorus (BP)@titanium dioxide (TiO_2_) hybrid photocatalysts

**DOI:** 10.1038/srep08691

**Published:** 2015-03-03

**Authors:** Hyun Uk Lee, Soon Chang Lee, Jonghan Won, Byung-Chul Son, Saehae Choi, Yooseok Kim, So Young Park, Hee-Sik Kim, Young-Chul Lee, Jouhahn Lee

**Affiliations:** 1 Division of Materials Science, Korea Basic Science Institute (KBSI), Daejeon 305-333, Republic of Korea; 2 Department of Applied Chemistry and Biological Engineering, Chungnam National University, Daejeon 305-764, Republic of Korea; 3 Division of Electron Microscopic Research, Korea Basic Science Institute (KBSI), Daejeon 305-333, Republic of Korea; 4 Korea Advanced Institute of Science and Technology (KAIST), Research Analysis Center, Daejeon 305-701, Republic of Korea; 5 Environmental Biotechnology Research Center, Korea Research Institute of Bioscience & Biotechnology (KRIBB), Daejeon 305-806, Republic of Korea; 6 Department of BioNano Technology, Gachon University, 1342 Seongnamdaero, Sujeong-gu, Seongnam-si, Gyeonggi-do 461-701, Republic of Korea

## Abstract

Over the past few decades, two-dimensional (2D) and layered materials have emerged as new fields. Due to the zero-band-gap nature of graphene and the low photocatalytic performance of MoS_2_, more advanced semiconducting 2D materials have been prompted. As a result, semiconductor black phosphorus (BP) is a derived cutting-edge post-graphene contender for nanoelectrical application, because of its direct-band-gap nature. For the first time, we report on robust BP@TiO_2_ hybrid photocatalysts offering enhanced photocatalytic performance under light irradiation in environmental and biomedical fields, with negligible affected on temperature and pH conditions, as compared with MoS_2_@TiO_2_ prepared by the identical synthesis method. Remarkably, in contrast to pure few layered BP, which, due to its intrinsic sensitivity to oxygen and humidity was readily dissolved after just several uses, the BP@TiO_2_ hybrid photocatalysts showed a ~92% photocatalytic activity after 15 runs. Thus, metal-oxide-stabilized BP photocatalysts can be practically applied as a promising alternative to graphene and MoS_2_.

Applications of nanoelectronics and optoelectronics as well as energy-related batteries and cells of two-dimensional (2D) and layered materials such as graphene with zero band gap^1^,^2^,^3^,^4^,^5^, not to mention the transition-metal dichalcogenide (TMDC) family (e.g., MoS_2_ with 1.23 eV–1.69 eV of indirect band-gap energy), are highly attractive research topics^3^,^4^. Although graphene has been widely and intensively developed for electronic and optical device applications^6^,^7^,^8^, limitations in its semi-metallic characteristics are emerging. These days, black phosphorus (BP) is a cutting-edge material due to its direct band-gap nature (i.e., ~2.0 eV for several layers and ~0.3 eV for the single layer, mainly depending on exfoliation of BP layers)^2^,^3^. Comparing the three main allotropes (white, red, and black) of phosphorus, the BP has merits including thermodynamic stability and insolubility in most solvents and lesser chemical reactivity and non-flammability^9^,^10^. As for crystalline structures, BP is present in three types, orthorhombic, rhombohedral, and cubic, along with the amorphous state^11^.

Several-layer BP crystals have been reported to demonstrate single-sheet-BP (i.e., phosphorene) practical feasibility^1^,^2^, showing, compared with the graphene- and MoS_2_-based alternatives^3^,^4^, higher carrier mobility and both *p*- and *n*-type configurations in field-effect transistor (FET) sensors^12^. Moreover, BP is used in lithium-ion batteries^9^,^13^ and thin-film solar cells^14^ that demonstrate large specific capacity and excellent cyclic performance, respectively which characteristics result in ~18% higher power-conversion efficiency than has been reported for trilayer graphene@ TMDCs solar cells.

Notwithstanding BP's superior optoelectronics, its photocatalytic performances in environmental and biomedical applications remain largely unexplored. Herein, taking into consideration semiconducting few layered BP and intercalation of TiO_2_ into a BP-layer system (BP@TiO_2_ hybrid) by one-pot reaction at room temperature, and comparing that system with an MoS_2_@TiO_2_ hybrid system fabricated by the identical preparation method, intriguingly novel photocatalytic performances over those of the traditional graphene-TiO_2_ hybrid photocatalytic composites^15^,^16^,^17^,^18^,^19^ were demonstrated.

## Results

### Morphological structures and elemental analysis

**Figure 1:**
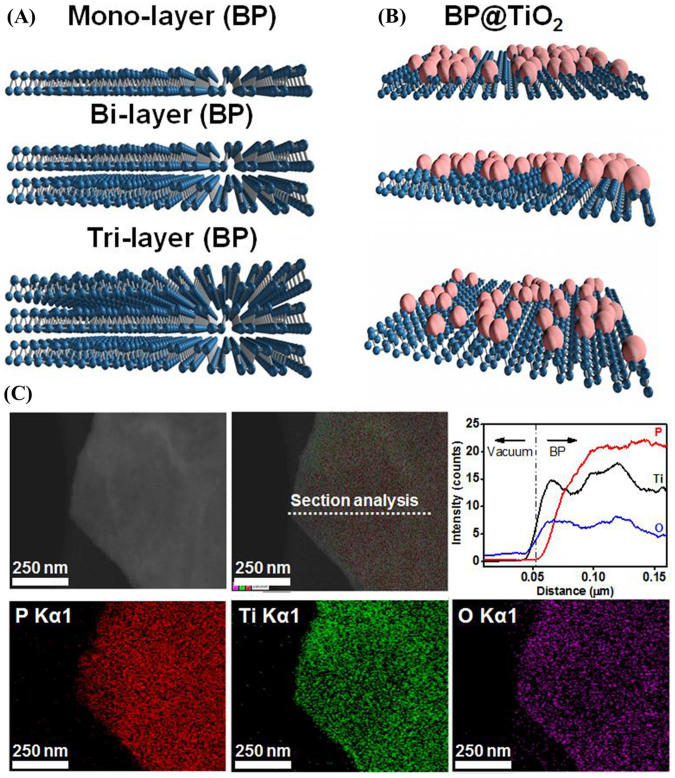
Modelled structures and elemental mapping analysis. Modelled mono-, bi-layer, and tri-layered BP structures (A), TiO_2_ substitution on BP structure (B), and line profile (top panel) and its elemental mapping (bottom panel) of P, Ti, and O elements in BP@TiO_2_ hybrid photocatalyst (C).

BP is generally known to have a puckered honeycomb structure with out-of-plane ridges in which each phosphorus atom is covalently linked to three neighboring single-layer phosphorus atoms while individual layer sheets are stacked vertically by van der Waals interaction^11^,^20^,^21^, as like the usual graphite pattern. The orthorhombic BP can be consisted of monolayer, bilayer and trilayer model structures (Fig. 1A). The well-defined crystalline morphology of few layered BP, which is prepared by an ultrasound-assisted delaminating process, displays crystal lattice fringes of ~2.5 Å (Supplementary Fig. 1) towards the (111) plane. A high-resolution transmission electron microscopy (HR-TEM) image of TiO_2_ substituted onto the BP surface reveals clearly distinct defects (Supplementary Fig. 1, yellow arrows)^22^, exactly corresponding to the modelling of the BP@TiO_2_ hybrid system (Fig. 1B), as looking like irregularly contained eggs (TiO_2_) in the tray. Reaction equations 1 and 2 show a possible BP@TiO_2_ formation mechanism. Ti(O_4_C_4_H_9_)_4_, when dropped into water, begins to rapidly hydrolyze, forming Ti(OH)_4_ on the few layered BP surface.
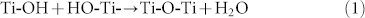

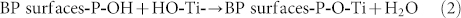
Then, Ti-OH was aggregated, forming Ti-O-Ti or Ti-O(H) bonds. Finally, an ultrasound irradiation process, the resultant ultrasonic cavitations creating a unique environment, induces a BP@TiO_2_ hybrid system with a better crystal structure^23^,^24^,^25^,^26^.

Elemental analyses of the BP@TiO_2_ hybrid photocatalysts entailed energy-dispersive X-ray (EDX) mapping of P, Ti, and O, where P is emitted from few layered BP, Ti indicates the presence of TiO_2-x_ (related titania compounds), and O originates from both few layered BP and TiO_2_ (Fig. 1C). Notably, Ti is uniformly distributed through the entire phosphorene surface. In the literature, TiO_2_ on graphene and MoS_2_, is deposited predominantly on the edges, due to the abundant functional groups of several-layer matrixes there^27^,^28^, leading to poor photocatalytic activity. As a result, it is needed additional carbon-coating steps to improve the uniformity of TiO_2_ nanoparticle distribution on the basal plane of MoS_2_^29^,^30^ or reduction in TiO_2_ band-gap energy^18^.

### X-ray diffraction (XRD) patterns and X-ray photoelectron spectra (XPS) analysis

**Figure 2:**
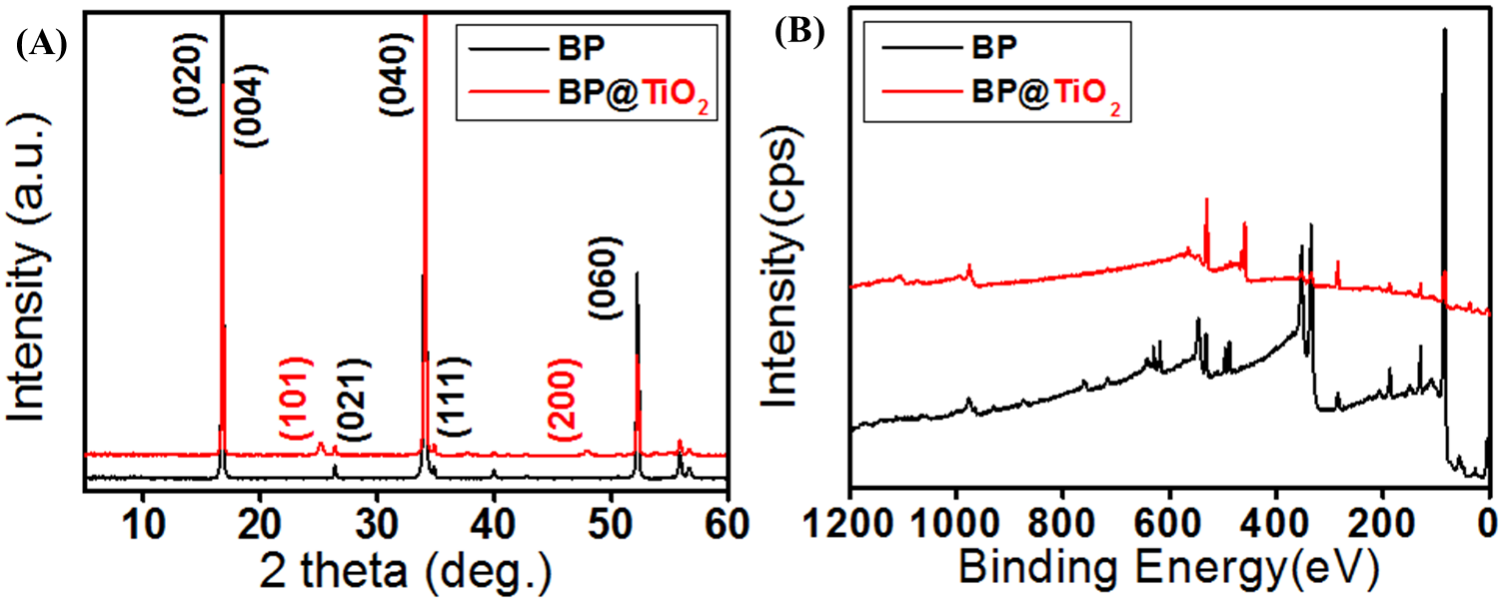
Crystalline structures and binding energy studies. X-ray diffraction (XRD) patterns (A) and scanning X-ray photoelectron spectra (XPS) results (B) for few layered BP and BP@TiO_2._ hybrid photocatalysts.

In order to confirm the crystalline structures and impurities of the novel photocatalysts, the X-ray diffraction (XRD) patterns (Fig. 2A) and X-ray photoelectron spectra (XPS) (Fig. 2B, Supplementary Fig. 2 and Table 1) were examined. The phases of as-synthesized TiO_2_ are composed of anatase/brookite at room temperature by sol-gel processing and ultrasound irradiation^31^. In particular, the synthesis of TiO_2_ nanoparticles with conjugation or substitution of other nanomaterials, the brookite phase in as-synthesized TiO_2_ was disappeared (data not shown)^32^. The XRD pattern of few layered BP was matched to the orthorhombic phase, with representative peaks of d_020_ = 5.243 Å, d_040_ = 2.6216 Å, and d_060_ = 1.7472 Å at 2θ = 16.897, 34.174, and 52.32, respectively (JCPDS no. 76–1957)^33^; the BP@TiO_2_ hybrid, meanwhile, formed anatase-structure peaks of d_020_ = 5.243Å, d_040_ = 2.6216 Å, d_040_ = 2.6216 Å, and d_060_ = 1.7472 Å at 2θ = 25.148, 37.671, 47.934, and 53.713, respectively (JCPDS no. 71–1168) but still retained the distinctive few layered BP peaks. A surface-elemental binding analysis of the XPS spectra of the BP@TiO_2_ hybrid photocatalyst confirmed, via general scanning, the presence of Ti2p, O1s, P2p, Au4f as a substrate, Sn3d (catalyst for BP preparation) and I3d (catalyst for BP preparation); in the pure few layered BP XPS spectra^34^ by contrast, Ti was not detected (Fig. 2B and Supplementary Table 1). XPS fitting of P2p and O1s was plotted to obtain specific information on the Ti-P and phosphated titania peaks at 128.6 and 134.4 eV, respectively (Supplementary Fig. 2). The phosphate titania exhibited a binding energy for P2p at 134.4 eV, indicating that the phosphorus in the sample exists in the pentavalent-oxidation state and apparently as P-O bonded species. The XPS profiles revealed, moreover, that the phosphorus ions at the surface of the BP@TiO_2_ exist in the P^5+^ and P^3+^ states.

### Optical properties of photocatalysts

In a further optical investigation of the few layered BP and BP@TiO_2_ hybrid photocatalysts, Ultraviolet-visible-near infrared (UV-Vis-NIR) reflection (%) and the absorbance spectra in few layered BP and BP@TiO_2_ hybrid photocatalysts showed most of the solar spectral regimes to be between the 250 nm and 1200 nm wavelengths, whereas the reference naked TiO_2_ was found mainly in the ultraviolet (UV)-absorbable region (Supplementary Fig. 3). These results indicated that few layered BP and BP@TiO_2_ hybrid photocatalysts are strongly responsive in visible-light regions, unlike naked TiO_2_, which is activated under UV-light irradiation. Photoluminescence (PL) spectra (Supplementary Fig. 4) at ~600 nm, for the few layered BP and BP@TiO_2_ hybrid photocatalysts at 350 nm excitation, showed mostly quenching phenomena, which was reflected in the fact that the BP@TiO_2_ hybrid photocatalyst was blue-shifted 40 nm, by which inhibition of electron-hole recombination and persistent production of powerful ·OH free radicals would be expected.

### Photocatalytic mechanism

**Figure 3:**
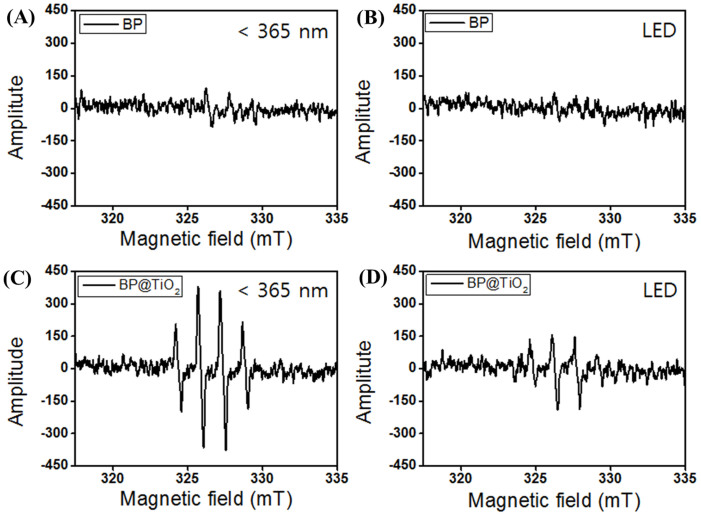
Detection of generated radicals. Electron spin resonance (ESR) spectra of few layered BP (A, B) and BP@TiO_2_ hybrid photocatalysts (C, D) at 365 nm and light-emitting diode (LED) irradiation, respectively.

Also, the electron spin resonance (ESR) spectra derived in the present study suggest spin-trapping method of photocatalysis for the few layered BP and BP@TiO_2_ hybrid photocatalysts (Fig. 3). After 5 mins' 365 nm irradiation of BP, a weak 1:2:2:1 pattern of ·OH free radicals was shown, but under light emitting diode (LED) irradiation, weaker and more negligible peaks were shown. Contrastingly, the BP@TiO_2_ hybrid photocatalyst exhibited strong ·OH free radical peaks under 365 nm UV-light irradiation, and even under LED irradiation, produced half-intensity ·OH free radical peaks^23^. According to the order in the ·OH free radical peak intensity for the few layered BP and BP@TiO_2_ hybrid photocatalysts, the order of the photocatalytic activity under visible-light irradiation was BP@TiO_2_ hybrid > few layered BP > TiO_2_. On this basis, the mechanism of the photocatalytic performance of the BP@TiO_2_ hybrid was derived (Supplementary Fig. 4). One fundamental assumption on the use the BP@TiO_2_ hybrid structure under visible-light irradiation is that the heterojunction between few layered BP and TiO_2_ will enhance the photo-generated electron-hole pair separation with electrons from the conduction band (CB) of the TiO_2_ injected into the few layered BP, while the hole trapped from valence band (VB) in the few layered BP and/or TiO_2_ will have longer lifetimes (Supplementary Fig. 5).

### Photocatalytic performances under UV- and visible light

**Figure 4:**
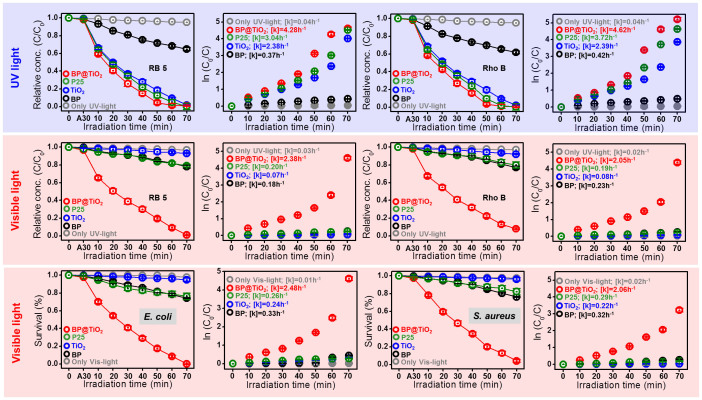
Photocatalytic performances. Relative concentrations and apparent reaction rate constants of RB 5 and Rho B of BP@TiO_2_ hybrid, P25, and few layered BP photocatalysts under UV- (A) and visible-light (B) irradiation, and antibacterial activities (C) and apparent reaction rate constants of *E. coli* and *S. aureus*.

Based on the measured intensities of the ·OH free radicals in the few layered BP and BP@TiO_2_ hybrid photocatalysts, the degradation of RB 5 (an anionic dye model) and Roh B (a cationic dye model) were tested. As plotted in Fig. 4A, during 70 min UV irradiation, the only-UV-light, few layered BP photocatalyst, naked TiO_2_ photocatalyst, P25, and BP@TiO_2_ hybrid photocatalysts showed apparent rate constants of 0.04, 0.37, 2.38, 3.04, 4.28 h^−1^ and 0.04, 0.42, 2.39, 3.72, 4.62 h^−1^ for RB 5 and Roh B, respectively. The UV-light condition of the BP@TiO_2_ hybrid photocatalyst afforded an apparent rate constant double that of naked TiO_2_ photocatalyst. By contrast, visible-light irradiation for the only-visible-light, few layered BP photocatalyst, naked TiO_2_ photocatalyst, P25, and BP@TiO_2_ hybrid photocatalyst showed 0.03, 0.18, 0.07, 0.20, 2.38 h^−1^ and 0.02, 0.23, 0.08, 0.19, 2.05 for RB 5 and Roh B, respectively (Fig. 4B), highlighting an almost ten-times-higher apparent rate constant for the BP@TiO_2_ hybrid system than for few layered BP photocatalyst. Few layered BP photocatalyst, meanwhile, showed an approximately three-fold-higher rate constant relative to that of naked TiO_2_ photocatalyst. As the reference experiments, in dark conditions, the photocatalytic activities in BP@TiO_2_, TiO_2_, P25, and few layered BP photocatalysts showed negligible performances (Supplementary Fig. 6). Next, the intermediate by-products concentration including dyes was measured by a total organic carbon (TOC) analyzer (Supplementary Fig. 7). After 70 min treatment, most of TOC values had reached ~92% with detoxified CO_2_ and H_2_O formation. As regards the recovery of the BP@TiO_2_ hybrid photocatalysts (Supplementary Fig. 8), significantly, even after the 15^th^ run, ~92.04% activity was maintained. Pure few layered BP photocatalyst, gradually decreasing after repeated runs, showed only ~30% photocatalytic activity: by the 8^th^ run, all of it had dissolved into the liquid state, thereby illustrating the difficult BP recovery. The robust stability of the BP@TiO_2_ hybrid system possibly is related to the substitution of Ti atoms into BP atomic lattices, which system effects resistance to humid and oxygen conditions^12^. Furthermore, similarly to the visible-light photocatalytic dye-degradation performance, the apparent rate constants of the antibacterial activities under the only-visible-light, few layered BP, TiO_2_, P25, and BP@TiO_2_ hybrid photocatalysts were 0.01, 0.33, 0.24, 0.26, and 2.48 h^−1^ for *Escherichia coli* (*E. coli*, as a gram negative species) and 0.02, 0.32, 0.22, 0.29, and 2.06 h^−1^ for *Staphylococcus aureus* (*S. aureus*, as a gram-positive species) (Fig. 4C). The colloidal behavior of the few layered BP and BP@TiO_2_ hybrid photocatalysts showed ~+12.73 mV and ~+3.18 mV zeta potentials in ethanol and ~−40.47 mV and ~−24.87 mV in distilled water, respectively. Clearly, in aqueous solution, few layered BP was hydrolyzed to form PO_4_^2−^ with a negatively charged surface, which resulted in a significantly diminished mechanistic stability. The BP@TiO_2_ hybrid system, though, was less hydrolyzed, and evidenced BP stabilization by displacement of TiO_2_ nanoparticles. In detail, at the empty sites of P atoms in few layered BP, Ti atoms were displaced at those positions in few layered BP <5 nm-sized TiO_2_ particles were substituted and formed onto the BP surface, uniformly and with negligible aggregations, finally producing BP@TiO_2_ hybrid photocatalysts and improving mechanical and photocatalytic stabilities in practical environmental and biomedical applications. According to zeta potential of BP@TiO_2_ hybrid photocatalysts, it exhibited negatively charged surface, but the adsorption amount of two different dyes displayed no marked difference. Thus, organic dye Roh B as a cationic dye resulted in slightly enhanced apparent rate of 4.60 h^−1^, compared to that of organic dye RB 5 (an anionic dye model).

## Discussion

In the diverse literature on graphene-based TiO_2_ hybrid photocatalytic activity enhancement^15^,^16^,^17^,^18^,^19^, graphene has played a multi-functional role. First of all, graphene enhances the visible-light absorbance region and the inhibition of electron-hole recombination by electron transfer from TiO_2_ to the graphene matrix as well as by reduction of TiO_2_ nanoparticle aggregation. Secondly, adsorption of targeting pollutants onto graphene@TiO_2_ hybrid photocatalysts accelerates degradation by TiO_2_ nanoparticles in graphene (oxide). Correspondingly, Lee *et al*. found that the wrapping of graphene sheets onto TiO_2_ nanoparticles decreased the band-gap energy in TiO_2_ for activation even under visible light^18^. For comparative purposes, in the present study, we investigated the photocatalytic activities of MoS_2_@TiO_2_ hybrid photocatalysts prepared according to the identical protocol. In the results, relatively very low apparent rate constants were obtained (Supplementary Fig. 9), resulting in approximately 20% dye degradation activity and antibacterial activity under visible-light irradiation. These poor photocatalytic activities can be attributed to the difficulty of uniformly decorating TiO_2_ nanoparticles on the MoS_2_ surface, despite the semiconducting action of the several-layer MoS_2_ sheets.

In summary, in order to effectively utilize unstable few layered BP in various applications, it is suggested that TiO_2_ nanoparticles can be substituted in the P atomic positions of BP with Ti by a simple method. The photocatalytic performances of BP@TiO_2_ hybrid photocatalysts in comparison with those of the semiconducting MoS_2_@TiO_2_ hybrid photocatalysts system were remarkable. Unlike MoS_2_, few layered BP can be mass-produced by phase transformation of red phosphorus^35^,^36^,^37^ or via a fast low-pressure transport route^38^. In the near future, studies on promising practical alternatives to graphene and MoS_2_, namely other unique metal oxides such as magnetic iron oxide (Fe_3_O_4_) and cerium oxide (CeO_2_), as deposited on few layered BP as a drug-delivery platform or therapeutic agent, currently are being planned, with particular emphasis on cellular targeting and smart retention time in the body.

## Methods

### Synthesis of BP@TiO_2_ hybrid photocatalysts

All of the reagents including commercial TiO_2_-P25 were of analytical grade (Sigma Aldrich, MO, USA) and were used without further purification. In the typical synthesis, black phosphorous (BP, 0.2 g, 6.4 mmol) was dispersed in a solution of anhydrous ethyl alcohol (400 mL) by high-intensity ultrasound irradiation for 2 h, to form few layered BP. 0.025 mol titanium isopropoxide dissolved ethyl alcohol solution (40 mL) was mixed with the BP-dispersed solution, 2 mL of which was then added drop-wise to deionized (DI) water with vigorous stirring for 10 min and finally treated by high-intensity ultrasound for 30 min. The dark-brown-colored BP@TiO_2_ hybrid solution was poured into the petri-dishes and dried on a plate at 60°C. The obtained product was dried under vacuum at room temperature (RT). Dispersion of BP and crystallization of TiO_2_ nanoparticles were performed under high-intensity ultrasound of 20 kHz frequency applied from the top of a polypropylene bottle reactor (~40 mL) using a Sonics and Materials VC750 ultrasonic generator. The electrical energy input was maintained at 100 W.

### Characterization

The crystalline structures of the BP@TiO_2_ hybrid samples were investigated with reference to X-ray diffraction (XRD; Rigaku RDA-cA X-ray diffractometer, Japan) patterns obtained by passing Cu Kα radiation through a nickel filter. The morphology of the BP@TiO_2_ particles was recorded by high-resolution transmission electron microscopy (HR-TEM; JEOL, JEM 2200, Japan). The samples prior to their analysis were placed on carbon coated copper grids and dried under ambient conditions. High-resolution X-ray photoelectron spectroscopy (HR-XPS) carried out using monochromatic Al Kα X-ray radiation (hν = 1486.6 eV) with a power of 120 W (Kratos Analytical, AXIS Nova, UK) was used to investigate the surface properties of the samples. The shift in the binding energy due to the relative surface charging was corrected according to the C1s level at 284.6 eV as an internal standard. Zeta potential measurements also were carried out, using a dynamic laser light scattering (DLS, Malvern Zetasizer NanoZS, USA) unit. A He-Cd laser (Kimmon, 1K, Japan) of 325 nm wavelength and 50 mW power was employed as an excitation source for photoluminescence (PL) measurements carried out using a spectrograph (*f* = 0.5 m, Acton Research Co., Spectrograph 500i, USA) with an intensified charge-coupled device (CCD, PI-MAX3, Princeton Instruments, IRY1024, USA). For free-radical detection by 5,5-dimethyl-1-pyrroline *N*-oxide (DMPO, 0.3 M in PBS buffer at pH 7.2, Sigma-Aldrich, USA) as a spin trap agent, an aliquot of sample (100 μL of 5 mg BP@TiO_2_ hybrid sample mixed with 300 μL DMPO solution) was filled into a capillary tube and directly irradiated with a UV (λ = 365 nm) or LED light (>400 nm) source for 5 min^39^,^40^, and the spectra were recorded by electron spin resonance (ESR) spectrometry (JES-FA200, JEOL, Japan). The specific ESR conditions were as follows: center field: 327 mT; powder: 1 mW; amplitude: 5.0 × 100; modulation width: 0.4 × 1; sweep width: 1 × 10; sweep time: 30 s.

### Measurement of photocatalytic and antibacterial activities

The photocatalytic degradations of reactive black 5 (RB 5; 3 mg/L, Sigma-Aldrich, USA) and rhodamine B (Rho B; 3 mg/L, pH 5.5, Sigma-Aldrich, USA) solutions by photocatalyst samples (1 g/L) were carried out under UV (source: 4 W, <365 nm, VSLAB VL-4CL, Korea) and visible-light (source: 150 W Xe lamp, λ > 420 nm, SCHOTT, USA) irradiation, and the absorbance of the solutions was measured using a UV-Vis-NIR spectrophotometer (Varian, Cary 5000, Australia) in the 200–800 nm wavelength region^41^,^42^. Before photocatalytic performances in all samples, adsorption-desorption equilibriums for 30 min were conducted. The concentrations of RB 5 and Rho B in the solutions after photoirradiation were measured from the absorbance peak intensities of the solutions at 598 and 555 nm, respectively. The changes in the concentration [ln(*C_0_*/*C*) = *kt*, where *k* is the apparent reaction rate constant, and *C_0_* and *C* are the initial and reaction concentrations, respectively, of Rho B] of the dye solution with reaction time for the samples were also investigated^41^,^42^. To demonstrate the stability of the photocatalysts, the BP@TiO_2_ hybrid was reused for the testing of other photocatalytic activities. The recycling tests for evaluation of the photocatalytic activity of BP@TiO_2_ hybrid photocatalysts were performed after washing the samples three times with DI water at 8500 rpm centrifugation and drying them in an oven for 6 h after each cycle. Additionally, the total organic carbon (TOC) of the solution was determined by a Shimadzu TOC-V analyzer (ELEMENTAR, vario TOC cub, Japan).

The antibacterial activities of the samples were evaluated according to the inhibition of gram-negative *Escherichia coli* (*E. coli*) and gram-positive *Staphylococcus aureus* (*S. aureus*) under visible-light irradiation^41^,^42^. Before these tests, all glassware and samples were sterilized by autoclaving at 120°C for 15 min. Bacterial cultures were grown in Luria-Bertain (LB) media overnight at 37°C with continuous shaking at ~200 rpm. The treated bacterial cells were diluted with DI water to a cell suspension of ~2 × 10^5^ colony-forming units (CFU/mL). Also, before photocatalytic performances in all samples, adsorption-desorption equilibriums for 30 min were conducted. The mass of the photocatalyst was adjusted to 25 μg/mL. The suspensions were stirred with a magnetic stirrer to prevent the samples from settling, and were then exposed to visible light for various irradiation times (0–70 min). Then, 1 mL of the suspension was sampled, added to the LB plate, and incubated overnight at 37°C. After incubation, the bacterial colonies were observed and quantified. As the reference experiments, dark experiments for all samples were conducted.

In measuring the photocatalytic and antimicrobial test performances, the data were averaged and expressed as mean ± standard deviations (SE). Each test was repeated up to five times. An analysis of variance (ANOVA) statistical analysis was performed, wherein *p*-values < 0.05 were considered significant.

## Author Contributions

H.U.L., Y.-C.L. and J.L. designed the project, organized the entire research. H.U.L., S.C.L., Y.-C.L., S.C., H.-S.K. and J.L. wrote the manuscript. H.U.L., S.C.L., Y.-C.L., J.W., Y.K. and S.Y.P. carried out the sample preparation and characterization. B.-S. performed the XPS analysis. H.U.L. and S.C. performed the photocatalytic performances. All authors discussed the results and commented on the manuscript.

## Supplementary Material

Supplementary Information Supplementary information
