# Supplementary material for: Stable semiconductor black phosphorus (BP)@titanium dioxide (TiO2) hybrid photocatalysts
Source: Sci Rep. 2015 Mar 3;5:8691. doi: 10.1038/srep08691 (PMC4346807; doi:10.1038/srep08691)
Supplement: Supplementary Information [file srep08691-s1.doc]

**Supplementary information**

**Stable semiconductor black phosphorus (BP)@titanium dioxide (TiO_2_) hybrid photocatalysts**

**Hyun Uk Lee,^1^ Soon Chang Lee,^2^ Jonghan Won,^3^ Byung-Chul Son,^4^ Saehae Choi,^5^ Yooseok Kim,^1^ So Young Park,^1^ Hee-Sik Kim,^5^ Young-Chul Lee,^6^**^*^ **and Jouhahn Lee^1^**^*^

^1^Division of Materials Science, Korea Basic Science Institute (KBSI), Daejeon 305-333, Republic of Korea, ^2^Department of Applied Chemistry and Biological Engineering, Chungnam National University, Daejeon 305-764, Republic of Korea, ^3^Division of Electron Microscopic Research, Korea Basic Science Institute (KBSI), Daejeon 305-333, Republic of Korea, ^4^Korea Advanced Institute of Science and Technology (KAIST), Research Analysis Center, Daejeon 305-701, Republic of Korea, ^5^Environmental Biotechnology Research Center, Korea Research Institute of Bioscience & Biotechnology (KRIBB), Daejeon 305-806, Republic of Korea, ^6^Department of BioNano Technology, Gachon University, 1342 Seongnamdaero, Sujeong-gu, Seongnam-si, Gyeonggi-do 461-701, Republic of Korea.

^*^Correspondence should be addressed to Y.-C.L.

E-mail:dreamdbs@gachon.ac.kr, Tel: 82-31-750-8751, Fax: 82-31-750-8774

^*^Correspondence should be addressed to J. Lee.

E-mail: jouhahn@kbsi.re.kr, Tel: 82-42-865-3613, Fax: 82-42-865-3610

**Table S1**

Obtained information summary of BP@TiO_2_ hybrid photocatalyst by integration of XPS

analysis.


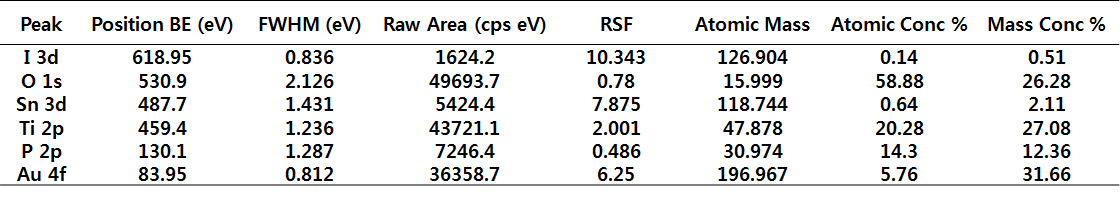


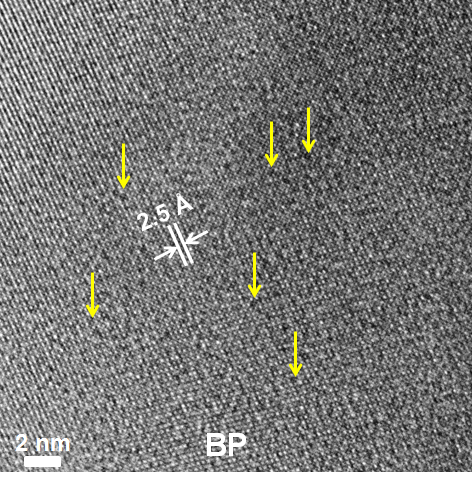


**Figure S1**. High resolution transmission electron microscopy (HR-TEM) image of

ultrasound-assisted delamination of BP. It is noted that yellow arrows mark the defects.


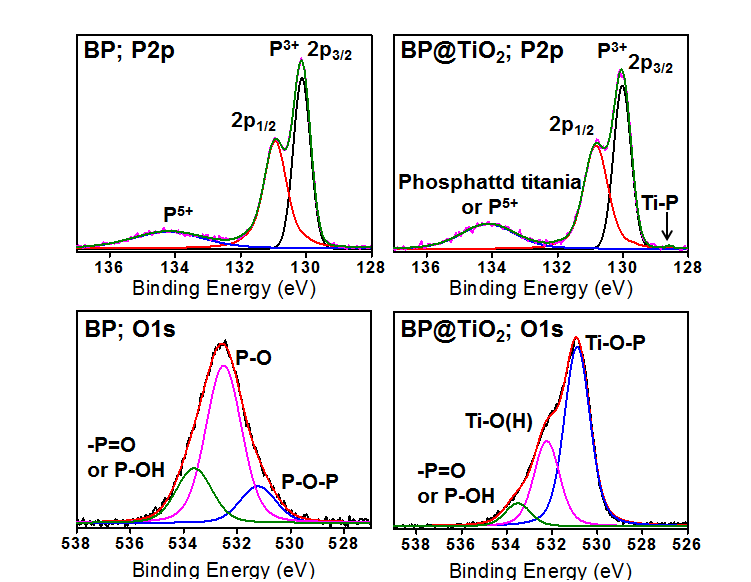


**A**

**C**

**B**

**D**

**Phosphated titania**

**Figure S2**. XPS spectra results of both BP (A,C) and BP@TiO_2_ hybrid photocatalyst (B,D).

P 2p of BP (A) and O 1s of BP (C) and P 2p of BP@TiO_2_ hybrid photocatalyst (B) and O 1s

of BP@TiO_2_ hybrid photocatalyst (D).

**
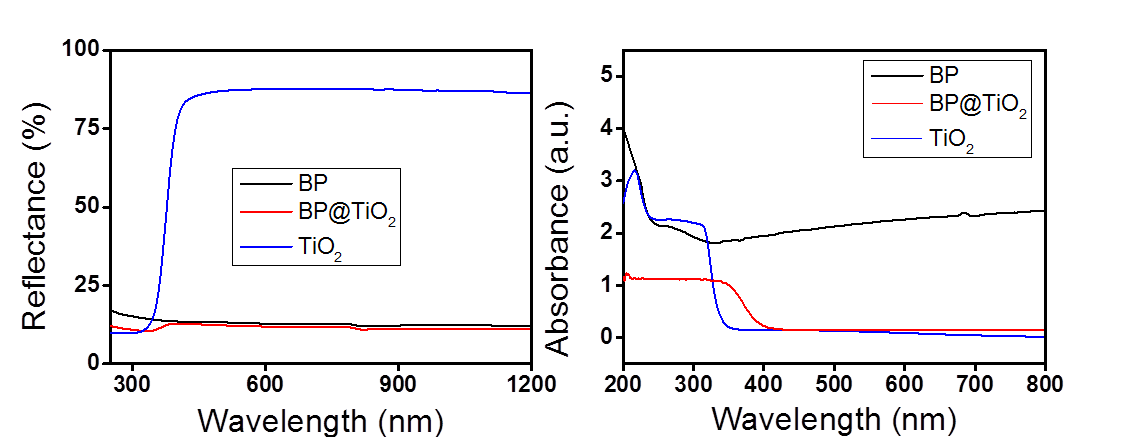
**

**A**

**B**

**Figure S3**. UV-Vis-NIR reflectance (A) and UV-Vis absorbance (B) spectra of BP,

BP@TiO_2_ hybrid photocatalyst, and TiO_2_ photocatalyst.

**
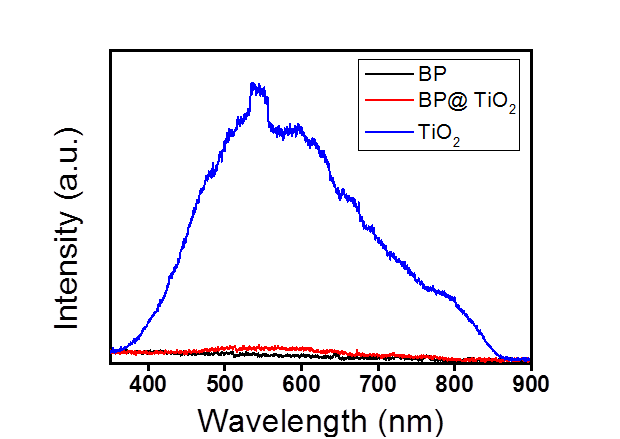
**

**Figure S4**. Photoluminescent (PL) quenching spectra of BP (black line), BP@TiO_2_ hybrid photocatalyst (red line), and TiO_2_ photocatalyst (blue line).

**
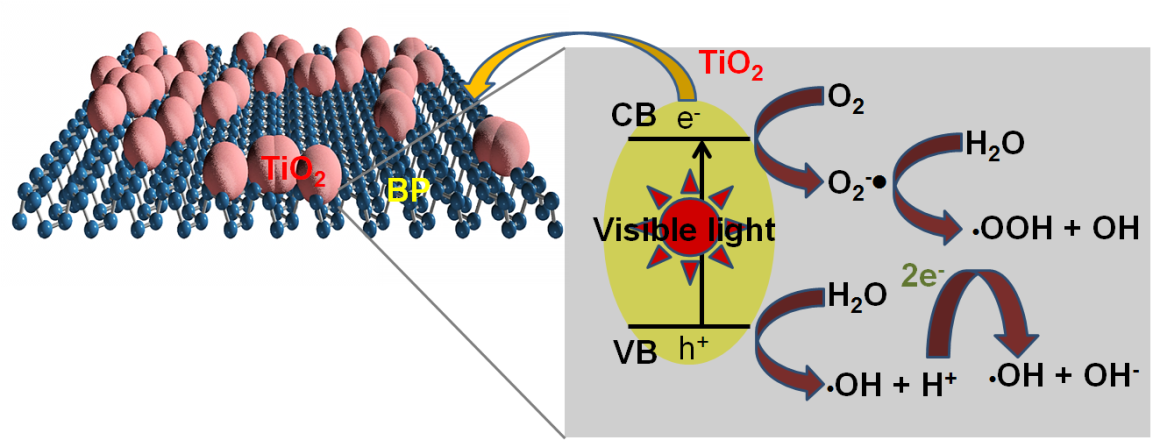
**

**Figure S5**. Suggested schematics of photocatalytic mechanism for BP@TiO_2_ hybrid photocatalyst under visible light irradiation.

**
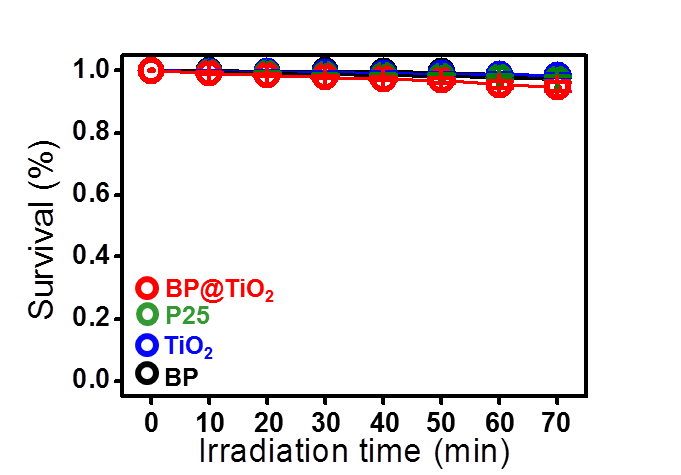
**

**Figure S6**. Antibacterial activities of BP@TiO_2_ hybrid, P25, and BP photocatalysts against *E. coli* in the dark condition.

**
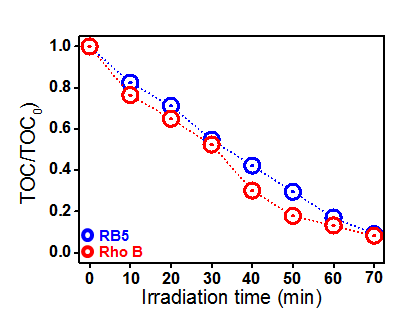
**

**Figure S7**. Total organic carbon (TOC) concentration results of RB 5 and Rho B by

BP@TiO_2_ hybrid photocatalyst under visible light irradiation.

**
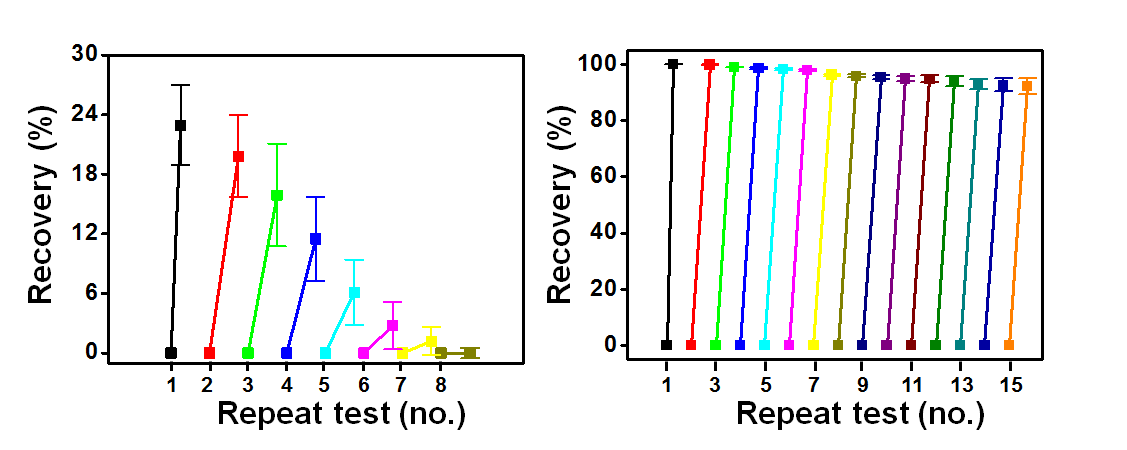
**

**A**

**B**

**Figure S8**. Recycle abilities of photocatalytic activities for RB 5 by BP photocatalyst (A) and BP@TiO_2_ hybrid photocatalysts (B) under visible light irradiation.

**
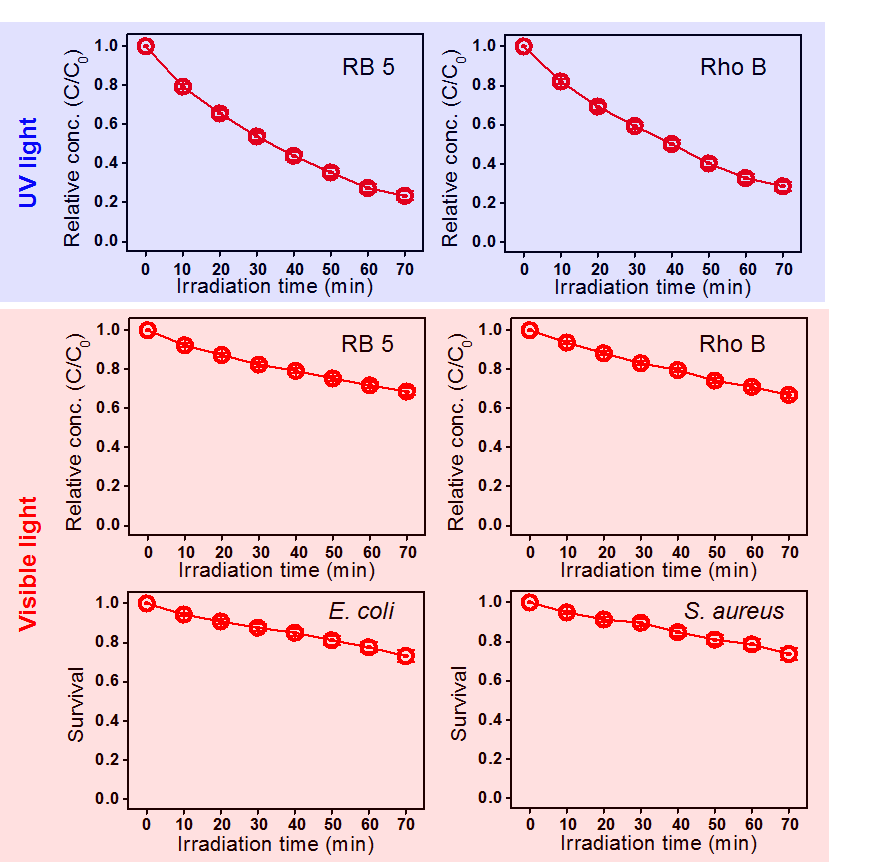
**

**A**

**B**

**Figure S9**. Photocatalytic performance of relative concentration RB 5 and Rho B, and antibacterial activities of *E. coli* and *S. aureus* by MoS_2_@TiO_2_ hybrid photocatalyst under visible light irradiation (B).
